# Topographical characterization of cone photoreceptors and the area centralis of the canine retina

**Published:** 2008-12-29

**Authors:** Freya M. Mowat, Simon M. Petersen-Jones, Helen Williamson, David L. Williams, Philip J. Luthert, Robin R. Ali, James W. Bainbridge

**Affiliations:** 1 Department of Genetics, University College London (UCL) Institute of Ophthalmology, London, UK; 2 Department of Small Animal Clinical Sciences, Michigan State University, East Lansing, MI; 3 Department of Clinical Veterinary Medicine, University of Cambridge, Cambridge, UK; 4 Department of Pathology, UCL Institute of Ophthalmology, London, UK

## Abstract

**Purpose:**

The canine is an important large animal model of human retinal genetic disorders. Studies of ganglion cell distribution in the canine retina have identified a visual streak of high density superior to the optic disc with a temporal area of peak density known as the area centralis. The topography of cone photoreceptors in the canine retina has not been characterized in detail, and in contrast to the macula in humans, the position of the area centralis in dogs is not apparent on clinical funduscopic examination. The purpose of this study was to define the location of the area centralis in the dog and to characterize in detail the topography of rod and cone photoreceptors within the area centralis. This will facilitate the investigation and treatment of retinal disease in the canine.

**Methods:**

We used peanut agglutinin, which labels cone matrix sheaths and antibodies against long/medium wavelength (L/M)- and short wavelength (S)-cone opsins, to stain retinal cryosections and flatmounts from beagle dogs. Retinas were imaged using differential interference contrast imaging, fluorescence, and confocal microscopy. Within the area centralis, rod and cone size and density were quantified, and the proportion of cones expressing each cone opsin subtype was calculated. Using a grid pattern of sampling in 9 retinal flatmounts, we investigated the distribution of cones throughout the retina to predict the location of the area centralis.

**Results:**

We identified the area centralis as the site of maximal density of rod and cone photoreceptor cells, which have a smaller inner segment cross-sectional area in this region. L/M opsin was expressed by the majority of cones in the retina, both within the area centralis and in the peripheral retina. Using the mean of cone density distribution from 9 retinas, we calculated that the area centralis is likely to be centered at a point 1.5 mm temporal and 0.6 mm superior to the optic disc. For clinical funduscopic examination, this represents 1.2 disc diameters temporal and 0.4 disc diameters superior to the optic disc.

**Conclusions:**

We have described the distribution of rods and cone subtypes within the canine retina and calculated a predictable location for the area centralis. These findings will facilitate the characterization and treatment of cone photoreceptor dystrophies in the dog.

## Introduction

The domestic dog, *Canis lupus familiaris*, is a genetically diverse species. Generations of line breeding have resulted in hereditary dystrophies manifesting in particular breeds. The dog is an important large animal model for human retinal disease. A range of gene defects responsible for inherited retinal diseases with close homology to human disorders have been identified [1–9]. Few inherited retinal defects have been characterized in other commonly used large animal models, such as the cat [10–12], and none have been described in the nonhuman primate. Recent clinical trials have reported successful use of gene replacement therapy for a hereditary form of childhood blindness, offering new hope for people with inherited retinal disease [13–15]. Safety and efficacy in Briard dogs with a similar genetic defect was central to the preclinical development of this work [16–18]. The canine eye enables the use of standard retinal surgical techniques for delivery of therapy into the subretinal space [19]. Vision can be assessed using behavioral analysis [20], and the longevity of the dog facilitates long-term evaluation [21,22].

Cone photoreceptor specific genetic mutations similar to those causing cone dystrophies in humans have been described in the canine species [2,23–25]. The development of new therapies for cone dystrophies will require a detailed understanding of the distribution of cones and cone subtypes in the canine retina. As therapy becomes more sophisticated, methods to target particular cell types within the eye will become more important, to increase the specificity and efficacy and limit potential side effects. The use of viral vectors with cone subtype specific promoters has been reported in the canine [26], further highlighting the need to establish the distribution of these cells within the large retinal area.

The mouse is the most commonly used small animal model for human genetic retinal disorders. The distribution of cone photoreceptors in this species has important differences to the human including the absence of a cone-rich region, differential distribution of cone subtypes in a superior/inferior gradient [27], and substantial coexpression of cone opsins [28]. Previous studies in the dog have examined the ganglion cell layer and have defined a visual streak of high ganglion cell density superior to the optic disc [29–33]. Within the visual streak, a temporal area of peak density is defined as the area centralis, which has important similarities to the human macula and is responsible for high visual acuity [29]. The location of the highest density of cones in the macula has well established anatomic landmarks in the human–the macular pigment and foveal avascular zone. In contrast, anatomic landmarks for identifying the area centralis in the dog have not been identified. The ability to predict confidently the location of highest cone density in the area centralis is therefore important when studying disorders involving cone photoreceptors and developing new therapeutic approaches.

The dog has two discrete cone subtypes [28]: the two cone opsins are sensitive to long/medium wavelength light (555 nm spectral sensitivity; red/green or L/M-opsin) and short wavelength light (429 nm spectral sensitivity; blue or S-opsin) [34,35]. Previous studies have examined cone density in retinal cross sections [26,36]. Unlike the detailed characterization of cone distribution in other species [28,37–39], cone subtype distribution in retinal flatmounts has only been examined qualitatively in dogs [30], and the location of the area centralis has not been accurately defined.

In this study we have performed a detailed quantitative evaluation of the distribution and density of the cone photoreceptors and subtypes in the beagle dog retina by immunohistochemical analysis. These findings will facilitate the experimental and preclinical development of new treatments for cone dystrophies.

## Methods

### Animals and tissue

Eyes for this study were from ophthalmoscopically normal research beagle dogs euthanized at Covance Research Laboratory Plc. (Harrogate, UK). Animals were obtained originally from Harlan UK Limited, Bicester, UK. We used the beagle exclusively for this study as it is of mesocephalic index, and a common research breed of dog. We examined a total of 18 eyes from 9 animals. Eyes were obtained immediately after euthanasia, and handled as described in the following section. Measurements of the zygomatic width and occiput to nose tip length were taken after euthanasia to calculate cephalic index as previously described [30] in addition to recording the age and sex of the animal. Age, sex, and skull measurements as previously described [30] were recorded at the time of euthanasia.

### Tissue preparation

The dorsal-most point of the limbus was marked to facilitate orientation of the isolated eyes, before enucleation. Approximately 0.5 ml of 2% paraformaldehyde (PFA) in phosphate buffered saline (PBS; Oxoid Ltd. Basingstoke UK; 8 g NaCl, 0.2 g KCl, 1.15 g Na_2_HPO_4_; 0.2 g KH_2_PO_4_), pH 7.4, was injected into the vitreal cavity via a pars plana injection site. Eyes were immersed in 2% PFA at 4 °C overnight. For flatmount analysis, retinas were dissected after removal of the lens and vitreous, and washed in PBS before immunohistochemistry. For retinal cryosections, the eyecup was cryoprotected in 20% sucrose for 12 h at 4 °C and embedded in optimal cutting temperature medium (Tissue Tek OCT medium; Raymond Lamb, Eastbourne, UK) before freezing. Sagittal 10 μm cryosections of the whole eyecup were taken in a superior-inferior orientation, facilitating comparison between the superior tapetal retina containing the visual streak and area centralis with the inferior non-tapetal retina on the same section.

### Immunohistochemistry

We studied retinal flatmounts and 10 μm retinal cryosections. Tissue was blocked overnight at 4 °C in a solution of PBS containing 1% normal goat serum, 5% BSA (BSA), and 0.1% Triton X-100. All antibodies were diluted in blocking solution. Table 1 lists the reagents and antibodies used in this study.

**Table 1:** Reagents and antibodies

**Reagent**	**Details**	**Source**	**Dilution**
Peanut agglutinin (PNA) [52]	biotin conjugated PNA	Vector Laboratories, Peterborough, UK	1: 1000
L/M opsin	polyclonal rabbit antibody	Chemicon Europe, Chandlers Ford, UK	1: 2000
S opsin	polyclonal rabbit antibody	Chemicon Europe, Chandlers Ford, UK	1: 2000

Manufacturers and dilutions of reagents used for PNA and opsin staining of retinal flatmounts and cryosections.

Retinal flatmounts were incubated with primary antibodies for 3 days at 4 °C with agitation. Retinal sections were incubated with primary antibodies overnight at 4 °C. We used all secondary antibodies at 1:1,000 dilutions (polyclonal goat antibodies, AlexaFluor conjugated; Invitrogen Molecular Probes, Paisley, UK). We incubated flatmounts with secondary antibody for 16 h in blocking solution at 4 °C with agitation and sections for 2 h at room temperature.

Flatmounts were mounted in aqueous fluorescent mounting medium (Dako Ltd., Ely, UK), and imaged as detailed in the next section. Retinal cryosections were counterstained using Hoechst 33342 nuclear counter stain, and mounted using fluorescent mounting medium.

### Imaging and analysis

#### Mapping the cone density of retinal flatmounts

For density mapping of flatmounts using peanut agglutinin (PNA) only, we quantified fluorescent microscopy images that were taken using a Leitz Diaplan fluorescent microscope (Leica Microsystems Ltd. Milton Keynes, UK) at 40X magnification in a grid pattern at 2 mm intervals throughout the retina, centering on the optic disc. Images were loaded unmodified into an image analysis program (Image Pro Plus, Media Cybernetics, Bethesda, MD). The total area of the image in mm^2^ was calculated by imaging a graticule (100 and 10 μm gradations) with the same objective lens and using the spatial calibration tool in the image analysis program. The total number of cones in the image was counted, and the number represented as a density per mm^2^. A total of 9 retinas from 5 animals were analyzed in this manner. Retinal area was calculated by scanning the flatmounts at high resolution and using the magic wand tool in the image analysis program to delineate the retinal area. One retina was imaged at 10X magnification with an Olympus FluoView 1000 confocal microscope (Olympus UK Ltd. Watford UK) with an automated stage. Multi-area time lapse was used to create a stitched image of a large portion of the retina including the visual streak and area centralis.

The whole optic nerve had been preserved during preparation in 7 of the 9 retinas. The optic nerve was imaged using a single x-y plane at 5X magnification on a Zeiss LSM 510 confocal (Carl Zeiss Ltd. Welwyn Garden City, UK) microscope. Images were loaded into an image analysis program (Image Pro Plus, Media Cybernetics, Media Cybernetics, Bethseda, MD), and a mean diameter for each optic nerve head was calculated by averaging 3 values. An average value for the optic nerve head diameter was calculated and used in subsequent calculations.

#### Differential interference contrast analysis of rod and cone inner segment size and density

We subsequently used 6 of the previously described 9 retinas for differential interference contrast (DIC) analysis. A Zeiss Axiophot fluorescence microscope (Carl Zeiss Ltd.) with a 100X DIC compatible lens was used. The rod and cone inner segments were used as the focal plane. Based on the analysis outlined in the previous section, the area centralis was identified; 3 images were taken from the retina in the area centralis and 3 images were taken from the inferior peripheral retina. DIC images were overlain with fluorescent PNA staining to allow positive identification of cone inner segments. Images were calibrated using a graticule image as outlined in the previous section. Images were cropped to 500 pixels square. The cross-sectional area of each cone inner segment within this field was measured using the image analysis program, and an average cross-sectional area per cone inner segment was calculated. In addition, a minimum of 5 rod inner segments per image were outlined, and an average cross-sectional area per rod inner segment was calculated. Using these data, we analyzed the large uncropped image for cone number, and cone and rod density.

#### Opsin subtype distribution

To map the opsin subtypes in retinal flatmounts, we examined flatmounts stained with PNA and either L/M or S cone opsin as detailed in the previous section. Confocal microscope z-stack projections were taken through the retinal inner and outer segments using a Zeiss LSM 510 confocal microscope at 63X magnification. Ten images were taken from each retina (3 retinas each for L/M and S opsin). Five images were taken at 0.5 mm intervals around the area centralis, and 5 images were taken at 0.5 mm intervals in the inferior peripheral retina, 10 mm inferior to the area centralis location. Images were loaded unmodified into an image analysis program; images were quantified for total cone number (PNA positive inner/outer segments) and opsin positive cones (PNA and opsin positive inner/outer segments). From these data, calculations were made for cone density per mm^2^, opsin positive cone density per mm^2^, and percentage opsin expression. An average value for the area centralis and inferior periphery was calculated for each eye and used in subsequent statistical analysis. To verify the flatmount data, we examined 10 μm retinal cryosections spanning the predicted area centralis location (1–2 mm temporal to the optic disc). Serial sections were stained with either L/M or S opsin antibody in addition to peanut agglutinin. Sections were viewed using a Leitz Diaplan microscope at 100X magnification. We counted entire PNA positive cone inner/outer segments and opsin positive inner/outer segments; the percentage of cone opsin positive cones was calculated. Three sections per eye were quantified; in each section, 3 high power fields (HPF) were counted in the area centralis and 3 HPF in the inferior periphery. A total of 3 eyes were examined. An average value per eye was calculated and used in subsequent statistical analysis.

### Statistical and mathematical analysis

An unpaired *t*-test with Welch’s correction was used to compare all data. Grid data from retinas stained with PNA were analyzed in MatLab R2007a (The MathWorks, Cambridge, UK). Data sets were translated to place the optic nerve head at the origin, and data were rotated, reflected, and overlaid to calculate a mean image.

## Results

We examined 18 retinas from 9 beagle dogs. Of these retinas, 15 were examined as flatmounts and 3 by retinal cryosection analysis. We recorded the age at euthanasia and cephalic index for each animal. We measured the retinal area for all flatmounts. A summary of these data are shown in Table 2.

**Table 2:** Summary of animal data and retinal flatmount area

	**n**	**minimum**	**maximum**	**mean**	**SEM**
Age (months)	9	8	15	10.6	1.1
Cephalic index	9	47.5	61.1	53.8	1.5
Retinal area (mm^2^)	15	706.2	837	746.8	11.4

Summary of data collected on age at euthanasia, cephalic index and measured retinal area for retinal flatmounts. Maximum and minimum values are represented with a mean value and Standard error of the mean (SEM).

### Rod and cone inner segment cross-sectional area and density

**Figure 1:**
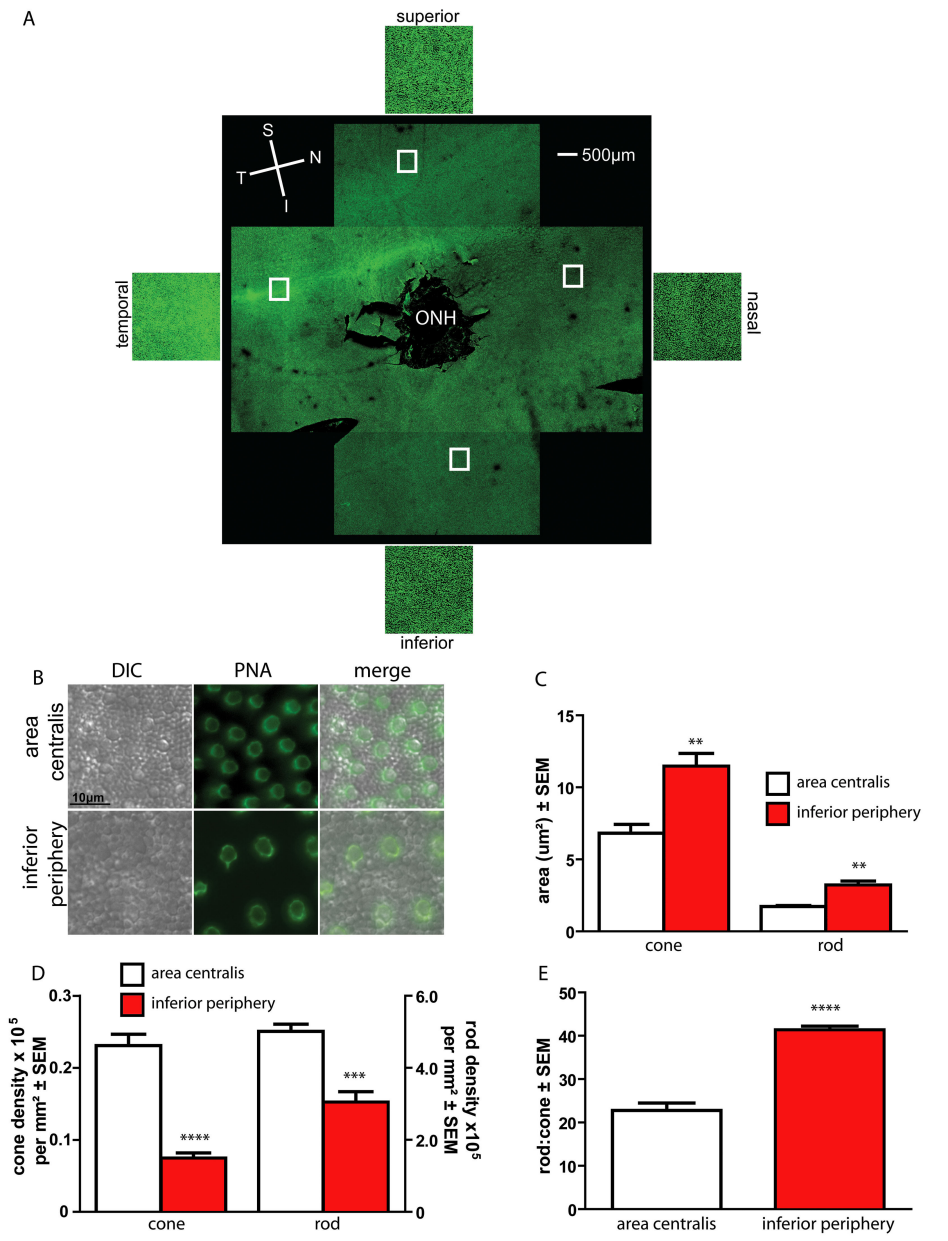
Comparison between rod and cone inner segment size, density, and ratio in the area centralis versus the inferior periphery. Six eyes from 3 animals were examined. On retinal flatmounts stained with peanut agglutinin (PNA; green) we identified a clear visual streak superior to the optic nerve, with a temporal area centralis. A representative retinal flatmount is shown in **A** illustrating a clear visual streak superior to the optic nerve head (ONH). Orientation is depicted: nasal (N), temporal (T), superior (S), and inferior (I), and representative magnified images from the highlighted areas of the retina are shown. The area centralis contained a higher density of smaller rods and cones than the inferior periphery. **B** shows representative differential interference contrast (DIC) images of flatmounts from the area centralis and the inferior periphery. PNA was used as a positive marker of the cone inner segment (green). Six areas per retina were examined (3 area centralis, 3 inferior periphery) and average calculations for the rod and cone inner segment cross sectional area and number were made. **C** shows that the inner segment area of both cones and rods was significantly smaller in the area centralis (cone: p=0.0028, rod: p=0.0034). The density of both rods and cones was significantly higher in the area centralis than in the periphery (**D**, cone: p<0.0001, rod: p<0.0005). Note the difference in scale of each y-axis. **E** shows the ratio between rods and cones in the areas of the retina; the ratio was significantly lower in the area centralis (p<0.0001). Using an unpaired *t* test, significant values are marked with asterisks **** p<0.0001, *** p<0.001, ** p<0.01. In all graphs, the inferior periphery bars are marked in red.

We compared the cross-sectional area and density of cone and rod photoreceptor inner segments in the area centralis with the inferior peripheral retina by quantifying DIC microscopy images of 6 retinal flatmounts from these areas (Figure 1). The focal plane of the DIC images was at the level of the rod and cone inner segment.

We identified the visual streak and peak area of cone density (area centralis) in each retinal flatmount using fluorescent labeling of cone matrix sheaths with peanut agglutinin (a representative low power image is shown in Figure 1A). We quantified both the inner segment cross-sectional area and the density of rods and cones using differential interference contrast images from the flatmounts (representative images are shown in Figure 1B). Both rod and cone inner segment cross-sectional area was significantly smaller in the area centralis than the inferior periphery (Figure 1C; cone: 6.82±0.62 μm^2^ area centralis, 11.46±0.90 μm^2^ inferior periphery, p=0.0028; rod: 1.73±0.06 μm^2^ area centralis, 3.22±0.28 μm^2^ inferior periphery, p=0.0034). Both cone and rod density was significantly higher in the area centralis than the inferior periphery (Figure 1D; cone: 23,080±1,593 per mm^2^ area centralis, 7,465±726 per mm^2^ inferior periphery, p<0.0001; rod: 501,000±20,180 per mm^2^ area centralis, 30,4800±28,700 per mm^2^ inferior periphery p<0.0005). Rod to cone ratio (Figure 1E) was significantly lower in the area centralis than the inferior periphery (rod: cone 22.8±1.7 area centralis, 41.4±0.9 inferior periphery p<0.0001) due to a combination of a smaller inner segment cross-sectional area and a higher density. We identified no areas where cone inner segments were present exclusively and rod inner segments were absent.

### Cone opsin subtype distribution

**Figure 2:**
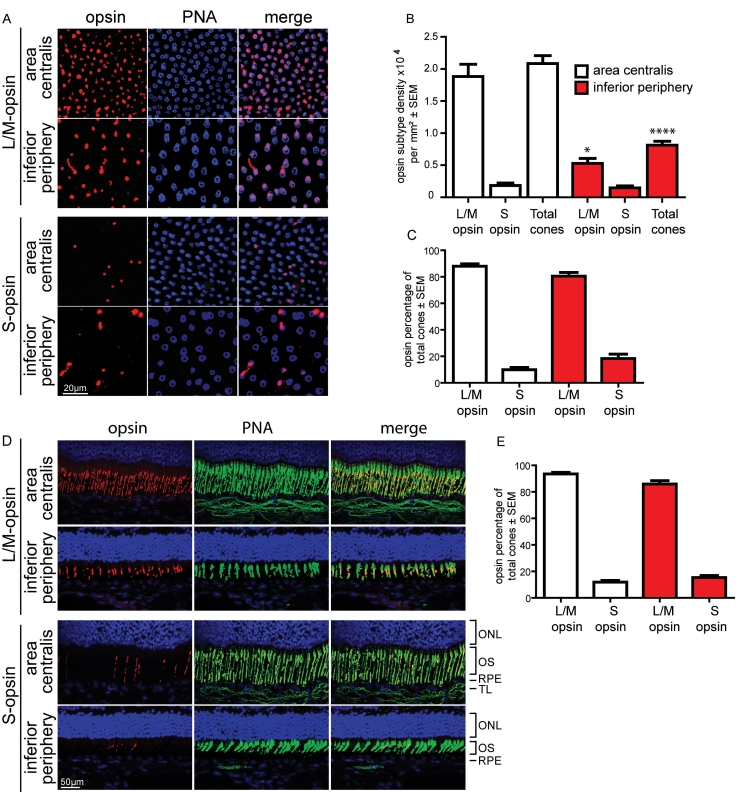
Comparison of opsin subtype distribution in the beagle retina. Nine eyes from 5 animals were examined. **A-C:** retinal flatmounts stained with peanut agglutinin (PNA; blue) and either long wavelength (L/M) or short wavelength (S) cone opsin (red) were analyzed. Representative images are shown in **A**. Absolute L/M cone density was significantly higher in the area centralis (**B**; p=0.0225), there was no difference in S cone density between the 2 areas examined (**B**; p=0.49). Quantification of L/M and S cones as a percentage of total cone number showed no statistical difference between the two areas examined (**C**; S cones p=0.16, L/M cones p=0.12). **D-E:** Serial 10 μm retinal cryosections were stained with PNA (green) and either L/M or S opsin (red) in a similar manner. Representative images are shown in **D**. The following areas in cross section are identified in **D:** outer nuclear layer (ONL), inner/outer segments (OS), retinal pigment epithelium (RPE), tapetum lucidum (TL) Nuclei are identified with Hoechst 33342 in blue. Analysis of cryosections for L/M and S cone percentage in the two areas examined showed no significant difference, supporting the flatmount data (**E**; S cones p=0.18, L/M cones p=0.11). Using an unpaired *t*-test, significant values are marked with asterisks **** p<0.0001, * p<0.05. In all graphs, the inferior periphery bars are marked in red.

We investigated the distribution of cone subtypes by immunohistochemistry using cone opsin specific antibodies in combination with peanut agglutinin in 6 retinal flatmounts (Figure 2). Confocal microscopy z-projection images were analyzed to identify the proportion of each cone subtype as a percentage of total cone number. In each retina we compared the area centralis with the inferior peripheral retina. Results were verified by immunohistochemistry of retinal cryosections in 3 eyes. We quantified each cone subtype in the area centralis and the inferior peripheral retina as a percentage of total cone number.

The majority of cones in the retinas expressed L/M opsin; S cones represented a small proportion of cones. In retinal flatmounts (representative images are shown in Figure 2A), absolute numbers of L/M cones were significantly higher in the area centralis (Figure 2B; density 18,820±1,913 per mm^2^ area centralis, 5,276±777 per mm^2^ inferior periphery p=0.0225), which closely matched the difference in total cone density in these areas (Figure 2B 20,850±1,220 per mm^2^ area centralis, 8,116±633 per mm^2^ inferior periphery, p<0.0001). The density of S cones was not significantly different between the two areas examined (Figure 2B; 1,842±371 per mm^2^ area centralis, 1,461±318 per mm^2^ inferior periphery, p=0.49). The proportion of S cones as a percentage of total cones was higher in the inferior periphery, but this difference was not significant (Figure 2C; 18.3±3.4% versus 9.9±1.7, p=0.16). The percentage of L/M cones was not significantly different between the inferior periphery and the area centralis (Figure 2C; 80.5±2.8% versus 88.0±1.9%, p=0.12). Calculations of cone subtype percentages from retinal cryosections confirmed the flatmount data (representative images are shown in Figure 2D); there was no significant difference in the percentage of either cone subtype between the area centralis and the inferior periphery (Figure 2E; L/M opsin: 93.6±1.2% area centralis, 86.0±2.5% inferior periphery p=0.11, S opsin: 11.9±1.3% area centralis, 15.4±1.5% inferior periphery, p=0.18).

### Predicting the location of the area centralis

We examined 9 retinal flatmounts from 5 beagle dogs. We took fluorescent microscopy images of peanut agglutinin staining in a grid pattern throughout the retina centering on the optic disc. Images were quantified for total cone number and the cone density per mm^2^ was calculated. Cone density was consistently highest in an area temporal to the optic disc (9/9 eyes) and lowest in the inferior periphery in the majority of retinas (5/9 eyes). A summary of cone density data for the area centralis and retinal periphery is presented in Table 3.

**Table 3:** Summary of cone grid density analysis

	**n**	**minimum**	**maximum**	**mean**	**SEM**
Area centralis cone density per mm^2^	9	20635	29848	26299	1138
Peripheral retina cone density per mm^2^	9	1872	4083	3053	259

A summary of cone density data in the area centralis and the retinal periphery from 9 retinal flatmounts analyzed in a 2 mm grid pattern. Maximum and minimum values are represented with a mean value and Standard error of the mean (SEM).

**Figure 3:**
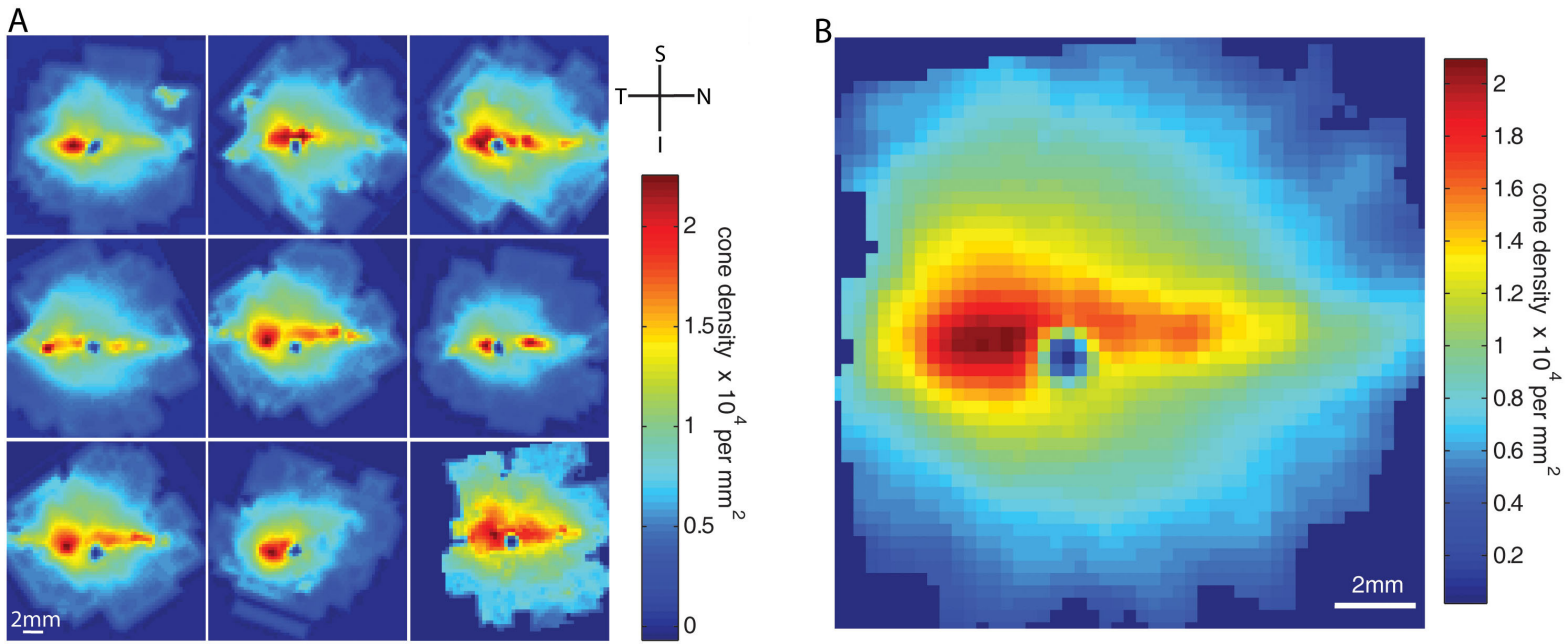
Cone density maps with correlation to predicted area centralis. Nine eyes from 5 animals were examined. **A** shows all 9 retinas used in analysis, demonstrating a marked visual streak of high cone density with an area centralis temporal to the optic nerve head. Data sets were reflected as necessary to represent all eyes as right. Orientation is depicted: nasal (N), temporal (T), superior (S), and inferior (I). **B** shows the average calculation from all 9 retinas demonstrating the mean density in the visual streak and a predictable location of the area centralis 0.4 optic nerve head (ONH) diameters superior and 1.1 ONH diameters temporal to the optic disc.

We analyzed and presented the data from each retina using the mathematical software MatLab. Grids were rotated and reflected to represent all eyes as right eyes. A clearly defined area of high cone density was evident superior to the optic disc in all retinas (Figure 3A). A pronounced visual streak extended nasally and temporally in 7 of 9 retinas, whereas a more moderate streak was present in 2 of 9 retinas, which did not extend nasally beyond the optic disc. The area centralis was identified as a peak of highest cone density located temporal to the optic disc in all 9 eyes. Those eyes with a moderate streak had a similar density and location of the area centralis to those with a pronounced streak.

Having measured the cone density in each of the 9 retinas examined, we calculated the mean distribution to predict the likely location of the area centralis in dogs of this breed (Figure 3B). The mean location of the area centralis from the 9 retinas was calculated as 0.6±0.1 mm superior to the optic disc, and 1.5±0.2 mm temporal to the optic disc. Using the mean of all 9 retinas, an area of high cone density around the visual streak with greater than 1.0x10^4^ cones per mm^2^ extended on average 6 mm nasally, 4.8 mm temporally and extended 4.2 mm superior to the optic disc. In the 7 retinas imaged, the mean optic nerve head diameter was 1.39±0.03mm. On average, the area centralis was located 1.1±0.3mm temporal and 0.4±0.1 optic nerve head diameters superior to the optic nerve head.

## Discussion

We have described in detail the distribution of the rod and cone photoreceptor subtypes and defined the likely location for the area centralis in the beagle dog. Both cone and rod densities are highest in the area centralis; cone and rod inner segments have a smaller cross-sectional area reflecting the higher density in this location compared with the peripheral retina. The majority of cones express L/M opsin. S opsin may be expressed in a higher proportion of cones in the peripheral retina than in the area centralis.

The area centralis contains a higher density of both rods and cones, and the inner segments of cells in this area have smaller cross-sectional areas. The inferior peripheral retina typically has the lowest cone density of the whole retina. It is likely therefore that the visual acuity is lowest in this location. Evidence from examination of visual fields from other species, including elasmobranchs [40] and artiodactyl mammals [41], have suggested that there is a close relationship between the location of high photoreceptor density visual fields and evolutionary adaptation. The low cone density in the inferior retina identified in this study may reflect evolutionary pressures: since the canine has few airborne predators or prey, visual acuity in the superior field may be of low priority. In the canine retina, L/M cone subtypes significantly outnumber S cones, particularly in the area centralis. L/M cone dominance enables better spatial and achromatic vision [42]. S cones comprise a relatively constant percentage of cones (9%–12% in the area centralis), although there is a suggestion that the percentage is higher in the inferior retina (15%–18%), consistent with the distribution of S cones found in the cat retina [43]. The low proportion of S cones compared with L/M cones may be a consequence of an evolutionary advantage of high resolution achromatic vision.

Results presented here are consistent with other investigations suggesting that the dog central retina is rod-dominant, even within the highest cone density region of the area centralis [44]. We provide further evidence to support the conclusion that the canine visual system is adapted for high performance in low light levels, but retains good function in higher light intensities [45]. We have determined that the cone photoreceptor area centralis is in a temporal location superior to the optic disc, within the tapetal fundus. The temporal location for this region of highest cone density may be relevant for enhancing binocular vision [29]. Previous studies have concluded that visual acuity in dogs is limited by the retina, not the optical properties of the eye, or higher processing in the brain [46]. The distribution of ganglion cells is well known, and here we provide evidence that the distribution of cone photoreceptors reflects that of ganglion cells. While we do not know the exact degree of convergence of cone photoreceptors to ganglion cells, the high peak concentration of cones in the area centralis is likely to support estimates of visual acuity of up to 20/65 previously described in the dog [47].

The distribution of ganglion cells within the visual streak of the canine retina has two distinct phenotypes: a “pronounced streak” and a “moderate streak” that may be breed-specific [29]. Our data suggest that cone distribution within the visual streak may have two similar phenotypes, with the majority of retinas from our beagle population demonstrating a pronounced streak phenotype. However, the visual streak cone distribution phenotype did not affect the location or the peak cone density of the area centralis. Other breeds of dog may also have variations in the visual streak cone distribution, but our data suggest that the location and cone density within the area centralis may be subject to less variation. By identifying the location of the area centralis in relation to the diameter of the optic nerve head, we provide clinically relevant information to those examining the canine fundus. Focal retinal thinning involving this location may affect high acuity color and bright light day vision. The density of rod photoreceptors in this location is also high, and degenerations affecting the area centralis may therefore have profound effects on vision as a whole. For this study we have developed a novel method to predict the location of the area centralis. Using detailed and quantitative imaging and analysis techniques, it may be possible to automate these methods to facilitate high throughput analysis.

Our findings provide new information on the comparative retinal anatomy of the dog and human. The dog has important advantages as a model of human disease over the mouse, which lacks an area of high density cones, although the dog is not directly comparable to the human which has a 100–150 μm diameter S cone-free macula [48]. In the dog, we found no peripheral ring of increased S cone density, which is present in humans [49]. We did not directly investigate coexpression of cone opsins; however, the proportions of photoreceptors expressing each cone opsin suggest that the extent of coexpression is low in the dog retina, which may be more analogous to the human in this respect. The proportion of S cones in the cone-rich area centralis is approximately 10%—similar to the human and macaque parafoveal area, which has a cone population comprising 8%–10% S cones [48,49].

We have identified several important differences between the dog and the human. Although the number of rods per cone was lower in the area centralis compared with the periphery, we identified no rod-free foveal area. This difference is a potential limitation of the dog as a model of human macular dystrophies. The data presented here suggest that the ratio between ganglion cells and cones is substantially lower in the dog than in the human or nonhuman primate. Humans and macaque monkeys have approximately 3 ganglion cells per foveal cone [50,51]. Previous studies of retinal flatmounts in the dog have estimated that the total ganglion cell number in the retina is 1.15×10^5^, and peak density in the area centralis is approximately 6,400–14,400 per mm^2^ [29]. Ganglion cells were not examined during this study, although the average density of cones in the area centralis was estimated at between 2 and 3×10^4^ per mm^2^. It is likely that more extensive summation of cone-derived signals occurs in dog visual processing in comparison with the primate.

We have illustrated that the visual streak extends nasally and temporally in a similar pattern to the tapetal region, and have defined a predicted location for the area centralis. Correlation of methods described with electrophysiological and behavioral assessments will provides a means of evaluating cone survival and function in disease models. The methods described will enhance the evaluation of the impact of new therapeutic interventions.

## References

[1] Ropstad EO, Narfstrom K, Lingaas F, Wiik C, Bruun A, Bjerkas E (2008). Functional and Structural Changes in the Retina of Wire-Haired Dachshunds with Early-Onset Cone-Rod Dystrophy.. Invest Ophthalmol Vis Sci.

[2] Mellersh CS, Boursnell ME, Pettitt L, Ryder EJ, Holmes NG, Grafham D, Forman OP, Sampson J, Barnett KC, Blanton S, Binns MM, Vaudin M (2006). Canine RPGRIP1 mutation establishes cone-rod dystrophy in miniature longhaired dachshunds as a homologue of human Leber congenital amaurosis.. Genomics.

[3] Zangerl B, Goldstein O, Philp AR, Lindauer SJ, Pearce-Kelling SE, Mullins RF, Graphodatsky AS, Ripoll D, Felix JS, Stone EM, Acland GM, Aguirre GD (2006). Identical mutation in a novel retinal gene causes progressive rod-cone degeneration in dogs and retinitis pigmentosa in humans.. Genomics.

[4] Kijas JW, Miller BJ, Pearce-Kelling SE, Aguirre GD, Acland GM (2003). Canine Models of Ocular Disease: Outcross Breedings Define a Dominant Disorder Present in the English Mastiff and Bull Mastiff Dog Breeds.. J Hered.

[5] Guziewicz KE, Zangerl B, Lindauer SJ, Mullins RF, Sandmeyer LS, Grahn BH, Stone EM, Acland GM, Aguirre GD (2007). Bestrophin gene mutations cause canine multifocal retinopathy: a novel animal model for best disease.. Invest Ophthalmol Vis Sci.

[6] Petersen-Jones SM, Entz DD, Sargan DR (1999). cGMP phosphodiesterase-alpha mutation causes progressive retinal atrophy in the Cardigan Welsh corgi dog.. Invest Ophthalmol Vis Sci.

[7] Aguirre GD, Baldwin V, Pearce-Kelling S, Narfström K, Ray K, Acland GM (1998). Congenital stationary night blindness in the dog: common mutation in the RPE65 gene indicates founder effect.. Mol Vis.

[8] Wiik AC, Wade C, Biagi T, Ropstad EO, Bjerkås E, Lindblad-Toh K, Lingaas F (2008). A deletion in nephronophthisis 4 NPHP4. is associated with recessive cone-rod dystrophy in standard wire-haired dachshund.. Genome Res.

[9] Beltran WA, Hammond P, Acland GM, Aguirre GD (2006). A frameshift mutation in RPGR exon ORF15 causes photoreceptor degeneration and inner retina remodeling in a model of X-linked retinitis pigmentosa.. Invest Ophthalmol Vis Sci.

[10] Menotti-Raymond M, David VA, Schäffer AA, Stephens R, Wells D, Kumar-Singh R, O'Brien SJ, Narfström K (2007). Mutation in CEP290 discovered for cat model of human retinal degeneration.. J Hered.

[11] Rah H, Maggs DJ, Blankenship TN, Narfstrom K, Lyons LA (2005). Early-onset, autosomal recessive, progressive retinal atrophy in Persian cats.. Invest Ophthalmol Vis Sci.

[12] Narfstrom K (1999). Hereditary and congenital ocular disease in the cat.. J Feline Med Surg.

[13] Bainbridge JW, Smith AJ, Barker SS, Robbie S, Henderson R, Balaggan K, Viswanathan A, Holder GE, Stockman A, Tyler N, Petersen-Jones S, Bhattacharya SS, Thrasher AJ, Fitzke FW, Carter BJ, Rubin GS, Moore AT, Ali RR (2008). Effect of Gene Therapy on Visual Function in Leber's Congenital Amaurosis.. N Engl J Med.

[14] Maguire AM, Simonelli F, Pierce EA, Pugh EN, Mingozzi F, Bennicelli J, Banfi S, Marshall KA, Testa F, Surace EM, Rossi S, Lyubarsky A, Arruda VR, Konkle B, Stone E, Sun J, Jacobs J, Dell'Osso L, Hertle R, Ma JX, Redmond TM, Zhu X, Hauck B, Zelenaia O, Shindler KS, Maguire MG, Wright JF, Volpe NJ, McDonnell JW, Auricchio A, High KA, Bennett J (2008). Safety and Efficacy of Gene Transfer for Leber's Congenital Amaurosis.. N Engl J Med.

[15] Bainbridge JWB, Tan MH, Ali RR (2006). Gene therapy progress and prospects: the eye.. Gene Ther.

[16] Le Meur G, Stieger K, Smith AJ, Weber M, Deschamps JY, Nivard D, Mendes-Madeira A, Provost N, Péréon Y, Cherel Y, Ali RR, Hamel C, Moullier P, Rolling F (2006). Restoration of vision in RPE65-deficient Briard dogs using an AAV serotype 4 vector that specifically targets the retinal pigmented epithelium.. Gene Ther.

[17] Narfström K, Bragadóttir R, Redmond TM, Rakoczy PE, van Veen T, Bruun A (2003). Functional and structural evaluation after AAV.RPE65 gene transfer in the canine model of Leber's congenital amaurosis.. Adv Exp Med Biol.

[18] Jacobson SG, Acland GM, Aguirre GD, Aleman TS, Schwartz SB, Cideciyan AV, Zeiss CJ, Komaromy AM, Kaushal S, Roman AJ, Windsor EA, Sumaroka A, Pearce-Kelling SE, Conlon TJ, Chiodo VA, Boye SL, Flotte TR, Maguire AM, Bennett J, Hauswirth WW (2006). Safety of recombinant adeno-associated virus type 2–RPE65 vector delivered by ocular subretinal injection.. Mol Ther.

[19] Komaromy AM, Varner SE (2006). de JE, Acland GM, Aguirre GD. Application of a new subretinal injection device in the dog.. Cell Transplant.

[20] Gearhart PM, Gearhart CC, Petersen-Jones SM (2008). A Novel Method for Objective Vision Testing in Canine Models of Inherited Retinal Disease.. Invest Ophthalmol Vis Sci.

[21] Narfström K, Vaegan, Katz M, Bragadottir R, Rakoczy EP, Seeliger M (2005). Assessment of Structure and Function Over a 3-year Period after Gene Transfer in RPE65−/− dogs.. Documenta Ophthalmologica..

[22] Narfström K, Seeliger M, Lai CM, Vaegan, Katz M, Rakoczy EP, Remé C (2008). Morphological aspects related to long-term functional improvement of the retina in the 4 years following rAAV-mediated gene transfer in the RPE65 null mutation dog.. Adv Exp Med Biol.

[23] Wiik AC, Wade C, Biagi T, Ropstad EO, Bjerkås E, Lindblad-Toh K, Lingaas F (2008). A deletion in nephronophthisis 4 (NPHP4) is associated with recessive cone-rod dystrophy in standard wire-haired dachshund.. Genome Res.

[24] Kijas JW, Zangerl B, Miller B, Nelson J, Kirkness EF, Aguirre GD, Acland GM (2004). Cloning of the canine ABCA4 gene and evaluation in canine cone-rod dystrophies and progressive retinal atrophies.. Mol Vis.

[25] Sidjanin DJ, Lowe JK, McElwee JL, Milne BS, Phippen TM, Sargan DR, Aguirre GD, Acland GM, Ostrander EA (2002). Canine CNGB3 mutations establish cone degeneration as orthologous to the human achromatopsia locus ACHM3.. Hum Mol Genet.

[26] Komaromy AM, Alexander JJ, Cooper AE, Chiodo VA, Acland GM, Hauswirth WW, Aguirre GD (2008). Targeting gene expression to cones with human cone opsin promoters in recombinant AAV.. Gene Ther.

[27] Szel A, Rohlich P, Mieziewska K, Aguirre G, vanVeen T (1993). Spatial and temporal differences between the expression of short- and middle-wave sensitive cone pigments in the mouse retina: a developmental study.. J Comp Neurol.

[28] Peichl L (2005). Diversity of mammalian photoreceptor properties: adaptations to habitat and lifestyle?. Anat Rec A Discov Mol Cell Evol Biol.

[29] Peichl L (1992). Topography of ganglion cells in the dog and wolf retina.. J Comp Neurol.

[30] McGreevy P, Grassi TD, Harman AM (2004). A strong correlation exists between the distribution of retinal ganglion cells and nose length in the dog.. Brain Behav Evol.

[31] Coli A, Marroni P (1996). Dog retinal ganglion cells: a morphological and morphometrical study in aging.. Anat Histol Embryol.

[32] Gonzalez-Soriano J, Rodriguez-Veiga E, Martinez-Sainz P, Mayayo-Vicente S, Marin-Garcia P (1995). A quantitative study of ganglion cells in the German shepherd dog retina.. Anat Histol Embryol.

[33] Krinke A, Schnider K, Lundbeck E, Krinke G (1981). Ganglionic cell distribution in the central area of the beagle dog retina.. Anat Histol Embryol.

[34] Neitz J, Geist T, Jacobs GH (1989). Color vision in the dog.. Vis Neurosci.

[35] Chiao CC, Vorobyev M, Cronin TW, Osorio D (2000). Spectral tuning of dichromats to natural scenes.. Vision Res.

[36] Koch SA, Rubin LF (1972). Distribution of cones in retina of the normal dog.. Am J Vet Res.

[37] Chandler MJ, Smith PJ, Samuelson DA, MacKay EO (1999). Photoreceptor density of the domestic pig retina.. Vet Ophthalmol.

[38] Kryger Z, Galli-Resta L, Jacobs GH, Reese BE (1998). The topography of rod and cone photoreceptors in the retina of the ground squirrel.. Vis Neurosci.

[39] Neitz M, Balding SD, McMahon C, Sjoberg SA, Neitz J (2006). Topography of long- and middle-wavelength sensitive cone opsin gene expression in human and Old World monkey retina.. Vis Neurosci.

[40] Litherland L, Collin SP (2008). Comparative visual function in elasmobranchs: spatial arrangement and ecological correlates of photoreceptor and ganglion cell distributions.. Vis Neurosci.

[41] Schiviz AN, Ruf T, Kuebber-Heiss A, Schubert C, Ahnelt PK (2008). Retinal cone topography of artiodactyl mammals: influence of body height and habitat.. J Comp Neurol.

[42] Chiao CC, Vorobyev M, Cronin TW, Osorio D (2000). Spectral tuning of dichromats to natural scenes.. Vision Res.

[43] Ahnelt PK, Kolb H (2000). The mammalian photoreceptor mosaic-adaptive design.. Prog Retin Eye Res.

[44] Kemp CM, Jacobson SG (1992). Rhodopsin levels in the central retinas of normal miniature poodles and those with progressive rod-cone degeneration.. Exp Eye Res.

[45] Miller PE, Murphy CJ (1995). Vision in dogs.. J Am Vet Med Assoc.

[46] Odom JV, Bromberg NM, Dawson WW (1983). Canine visual acuity: retinal and cortical field potentials evoked by pattern stimulation.. Am J Physiol.

[47] Murphy CJ, Mutti DO, Zadnik K, Ver HJ (1997). Effect of optical defocus on visual acuity in dogs.. Am J Vet Res.

[48] Bumsted K, Hendrickson A (1999). Distribution and development of short-wavelength cones differ between Macaca monkey and human fovea.. J Comp Neurol.

[49] Curcio CA, Allen KA, Sloan KR, Lerea CL, Hurley JB, Klock IB, Milam AH (1991). Distribution and morphology of human cone photoreceptors stained with anti-blue opsin.. J Comp Neurol.

[50] Harman A, Abrahams B, Moore S, Hoskins R (2000). Neuronal density in the human retinal ganglion cell layer from 16–77 years.. Anat Rec.

[51] Sjostrand J, Conradi N, Klaren L (1994). How many ganglion cells are there to a foveal cone? A stereologic analysis of the quantitative relationship between cone and ganglion cells in one normal human fovea.. Graefes Arch Clin Exp Ophthalmol.

[52] Long KO, Aguirre GD (1991). The cone matrix sheath in the normal and diseased retina: cytochemical and biochemical studies of peanut agglutinin-binding proteins in cone and rod-cone degeneration.. Exp Eye Res.

